# Serum geoepidemiology of leprosy biomarkers in a city-wide COVID-19 survey in Brazil

**DOI:** 10.1186/s12879-025-12483-0

**Published:** 2026-01-07

**Authors:** Filipe Rocha Lima, Mateus Mendonça Ramos Simões, Bruno Vitiritti, Cláudia Maria Lincoln Silva, Natália Aparecida de Paula, Vanderson Mayron Granemann Antunes, Josafá Gonçalves Barreto, Fernando Bellissimo-Rodrigues, Rodrigo de Carvalho Santana, Marco Andrey Cipriani Frade

**Affiliations:** 1 Laboratory for Skin Studies and Alternative Models, Ribeirão Preto Medical School, University of São Paulo, Ribeirão Preto, São Paulo Brazil; 2 Dermatology Division, Department of Internal Medicine, Clinical Hospital of the Ribeirão Preto Medical School, National Referral Center for Sanitary Dermatology and Leprosy, University of São Paulo, São Paulo, Brazil; 3 Department of Biochemistry and Immunology, Ribeirão Preto Medical School, University of São Paulo, Ribeirão Preto, São Paulo Brazil; 4 Spatial Epidemiology Laboratory, Federal University of Pará, Castanhal, Pará Brazil; 5 Department of Social Medicine, Ribeirão Preto Medical School, University of São Paulo, Ribeirão Preto, São Paulo Brazil; 6 Division of Infectious and Tropical Diseases, Department of Internal Medicine, Clinical Hospital of the Ribeirão Preto Medical School, University of São Paulo, Ribeirão Preto, São Paulo Brazil

**Keywords:** Leprosy, Surveillance, Antibodies, Serological test, Biomarkers, Georeferencing

## Abstract

**Background:**

COVID-19 has created a significant global health emergency and triggered numerous seroepidemiological field tracing initiatives. The use of these samples becomes a timely tool for intensifying active case finding and early diagnosis of leprosy. This study aimed to conduct a seroepidemiological evaluation using leprosy biomarker antibodies against the Mce1A and PGL-I antigens, and to analyze the spatial distribution of both actively detected cases and antibody levels, thereby characterizing the geoepidemiological pattern of bacillary circulation, combined with case-screening strategies.

**Methods:**

A cross-sectional and geoepidemiological study was carried out using the biorepository of samples from the COVID-19 serosurvey in a municipality in southeastern Brazil. Screening diagnosis using LSQ and the artificial intelligence system MaLeSQs^®^ was applied to investigate neurodermatological signs and symptoms of leprosy (*n* = 224). IgA, IgM, and IgG anti-Mce1A and IgM anti-PGL-I antibodies were measured using indirect ELISA (*n* = 195). Georeferencing was employed to create the distribution maps of individuals within the municipality. Global spatial autocorrelation analysis was performed and applied to the serological scores.

**Results:**

The responses to the clinical questionnaire reported the predominance of neurological signs and symptoms. Twelve new cases were diagnosed (32.4%), and the detection rate in the population sample evaluated was 6.15%. The IgA anti-Mce1A ELISA showed the highest seropositivity (55.3%), the highest rates [median = 0.93 (IQR = 0.59–1.41)]. The IgM and IgG anti-Mce1A serology showed higher rates (*P* < 0.0001) as compared to the anti-PGL-I serology. The overlap of positivity with the antibodies tested highlights the greater involvement of double or triple positives when Mce1A serology was used. IgM anti-Mce1A serology was positive in 66.7% [8/12 cases; median = 1.25 (IQR = 0.70–1.68)] of new cases detected. IgM anti-Mce1A showed the best serological performance, and its combination with MaLeSQs^®^ (OR condition) achieved 100% sensitivity and NPV, with 68% specificity. Serology with the IgA antibody presented the highest rates in georeferencing analysis and served as an alert for contact with the bacillus. The sociodemographic variables tested did not exhibit statistical difference in spatial autocorrelation (*p* > 0.05), indicating the absence of a spatially clustered pattern for serological values in the analyzed territory.

**Conclusion:**

These findings identify IgM anti-Mce1A and MaLeSQs^®^ as key tools to strengthen leprosy case-finding and screening efficiency. The study also revealed a diffuse pattern of transmission and exposure within the municipality and highlights the value of integrating serological biomarkers and digital diagnostic platforms to support earlier detection of leprosy.

**Supplementary Information:**

The online version contains supplementary material available at 10.1186/s12879-025-12483-0.

## Background

Leprosy is an infectious disease with a chronic course that can be treated with multidrug therapy (MDT) and whose etiological agents are the bacilli *Mycobacterium leprae* (*M. leprae*) and *Mycobacterium lepromatosis* [1]. Leprosy is transmitted through contact with the infected patient through the nasal and oral mucosa. The bacillus primarily affects the skin and peripheral nerves, and can lead to severe sequelae if left untreated, highlighting the importance of early diagnosis and therapeutic intervention [2, 3]. The epidemiological report released in 2023 by the World Health Organization (WHO) recorded 182,815 new cases of leprosy worldwide, with 13.6% of these cases occurring in the Americas. Notably, more than 90% of cases in the Americas were reported in Brazil [4]. The data demonstrated a reduction of 37% in the detection of new cases post-pandemic compared to 2019, as a result of the impact of the pandemic caused by Severe Acute Respiratory Syndrome Coronavirus 2 (SARS-CoV-2), the pathogenic agent of respiratory disease called Coronavirus Disease-19 (COVID-19) that has been spreading globally since December 2019 [5].

The COVID-19 pandemic had a direct impact on the diagnosis and monitoring of leprosy cases. The actions developed to control COVID-19 made it difficult for leprosy patients to access health services, necessitating the intensification of strategies for actively searching for new cases and early diagnosis [6]. Socioeconomic conditions such as social distancing, measures to restrict urban mobility, fear of infection, and reduced access to health services during the pandemic can drastically affect diagnosis, treatment, and control of leprosy, as high proportions of multibacillary (MB) leprosy cases, individuals diagnosed with a physical disability, and a poor treatment rate [7].

The detection of *M. leprae* infection, followed by effective intervention, is considered a vital component of strategies aimed at reducing leprosy transmission [3]. Immunoassays can quantitatively reveal the concentration of circulating antibodies and provide information on exposure to the pathogen [8]. The most widely used serology seeks the identification of IgM anti-phenolic glycolipid-I (APGL-I). High titers of IgM APGL-I of *M. leprae* are associated with dissemination and progressive infections by the bacillus, making the test more likely to be positive in cases with high bacillary loads (MB). The indirect enzyme-linked immunosorbent assay (ELISA) test is used to detect antibodies and is primarily valuable for diagnosing MB cases; however, its value in detecting paucibacillary (PB) patients is limited [9–11]. In hyperendemic regions for leprosy, more than 50% of individuals may present positive APGL-I responses [12]. However, some of these people with a positive antibody titer will never develop leprosy, even though positive APGL-I serology is a risk factor for the development of leprosy [13, 14].

Before the decoding of the *M. leprae* genome, the availability of new antigens was limited mainly because the bacillus was not cultivable in vitro [8]. The recombinant protein, isolated based on the mammalian cell entry 1 A (*mce1A*) gene, was shown to promote internalization and survival in macrophages [15], nasal epithelial cells, bronchial epithelial cells, dermal fibroblasts, microvascular endothelial cells, and human keratinocytes [16]. Therefore, the *mce1A* gene product may mediate the entry of *M. leprae* into respiratory epithelial cells, which are its natural target cells, and this may be the primary mode of transmission [16, 17]. Being a target molecule in the initial process of infection and the role of the Mce protein complex associated with lipid transport, modulation of host cell signaling, cell wall homeostasis, and cell membrane remodeling [18, 19]. Thus, the use of the recombinant Mce1A protein has already been applied as an immunoassay proposal for the detection of immunoglobulins IgA, IgM, and IgG [20–23]. The results found suggest that the proposed serological test can be used as an easy, non-invasive and low-cost method to support the diagnosis and monitoring of leprosy, considering the low seropositivity presented by paucibacillary (PB) patients and household contacts (HHC) when using other serological tests such as APGL-I serology for IgM detection and tests using a recombinant fusion protein (LID-1) [20–22].

Thus, to contribute to the clarification of the disease and the tracking of cases, facilitating the spatial mapping of priority clusters, a geoepidemiological study using serology was carried out with the biorepository of samples from the COVID-19 serosurvey in a municipality in southeastern Brazil, which was considered highly endemic for leprosy in 2022. This study aimed to evaluate leprosy biomarker antibodies and to analyze the spatial distribution of both actively detected cases and antibody levels, combined with case-screening strategies. Thus, to block the transmission chain of the bacillus, with diagnosis based on the antibody response to the Mce1A and PGL-I antigens, as well as the signs and symptoms associated with leprosy disease.

## Methods

### Study setting and design

This investigation is a cross-sectional study and geopidemiological survey. This bidirectional study used retrospectively collected specimens from the COVID-19 serosurvey for new serological assays to identify leprosy biomarkers, and additionally included the prospective recruitment of suspected cases for clinical neurodermatological evaluation for leprosy, combined with questionnaire on case screening strategies. The study was developed at the Laboratory of Skin Studies and Alternative Models of the Ribeirão Preto School of Medicine of the University of São Paulo (USP) using the sample biorepository “SARS-CoV-2 epidemiological survey in the population of Ribeirão Preto” [24, 25]. The samples used in the COVID-19 survey were collected from May 1 to 3 and June 11 to 14, 2020, in Ribeirão Preto, a medium-sized city in the state of São Paulo in southeastern Brazil, which in 2020 had an estimated population of 706,552 inhabitants [26].

The city of Ribeirão Preto is divided into five health districts (North, East, West, Central, and South). Census sectors were delineated within each health district, and households were selected for data collection within these sectors. The drawing of the sectors considered the respective São Paulo Social Vulnerability Indices [27]. In each household included in the study, only one person was drawn to represent the household and obtain a blood sample. The sampling plan was structured to represent the diversity of the population in terms of sex, age, skin color, education, socioeconomic stratum, and place of residence. Fifteen specifically trained teams visited 90 index addresses drawn in the five health districts of the city. Each index address represented a cluster of eight households, totaling 720 households to be approached. A stratified sampling plan was considered, in which each of these five regions comprises a stratum. Estimating a seroprevalence during the period for SARS-CoV-2 of 20%, with a confidence coefficient of 95%, and a sampling error of 3.0%. The analyses presented were entirely designed and conducted for the leprosy study, which used only stored serum and contact information from the prior COVID-19 survey as its starting point for the new serological analyses of leprosy biomarkers.

### Study population

**Fig. 1:**
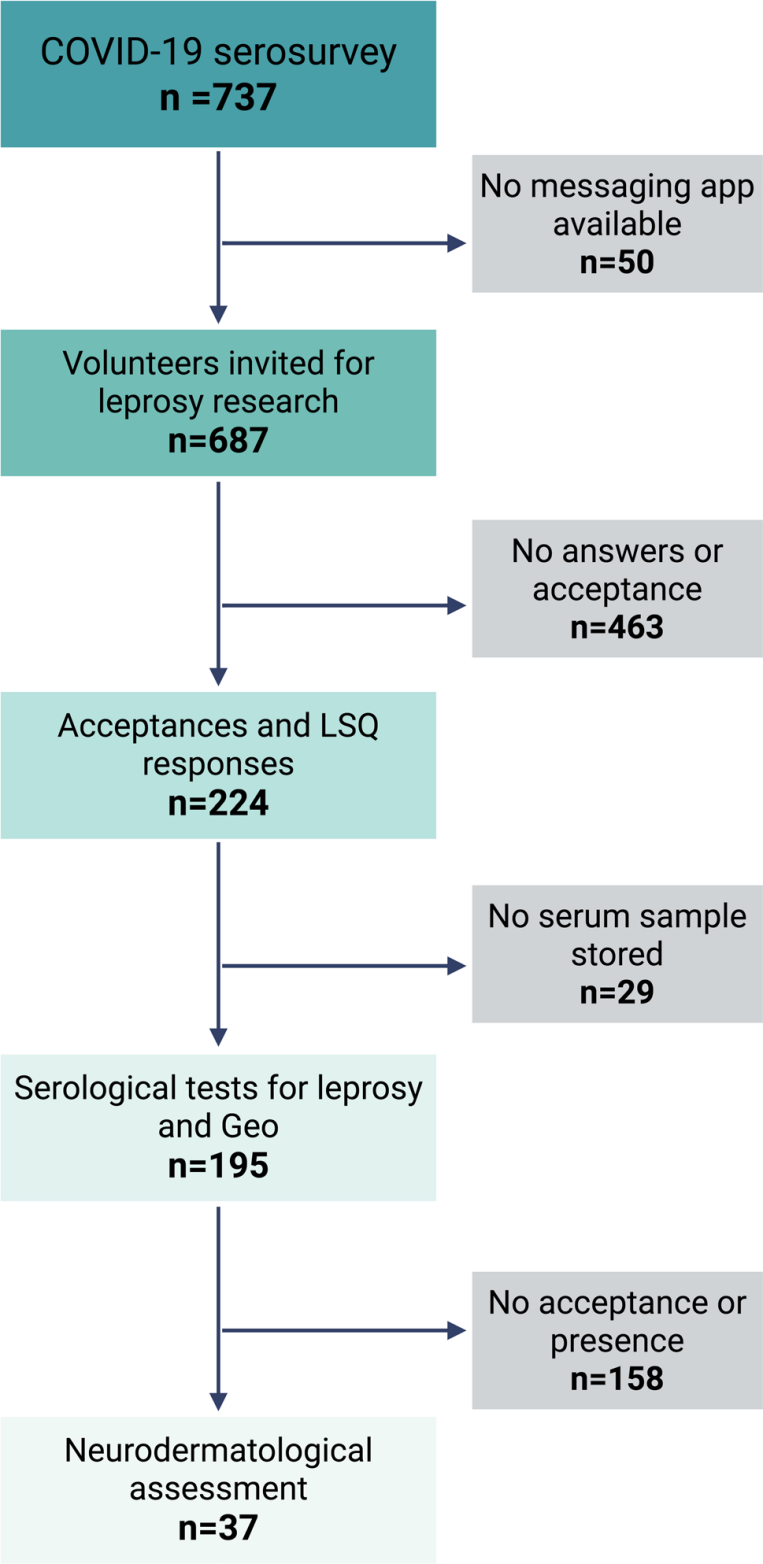
Flowchart of study population selection. The COVID-19 serological survey included 737 individuals, of whom 687 could be contacted via a messaging app. Among these, 224 agreed to participate in the leprosy biomarker study; serum samples were available for 195 participants, and 37 of them attended the clinical evaluation. GEO: georeferencing. Created in BioRender. Lima, F. (2026) https://BioRender.com/zggmras

In 2021, individuals from the COVID-19 survey (*N* = 737) were invited to participate in the research to identify serological biomarkers for leprosy through a digital form and by contact via a communication application. No financial or non-financial incentives were provided. Participation in the survey was entirely voluntary. Of these, *n* = 687 had the WhatsApp messaging application. As communication was virtual, lack of access to a messaging application was an exclusion criterion. The automatic messages were sent to study participants using a Python 3.9.12 script in the Jupyter Notebook environment, which utilized the Pandas 1.4.2 and Selenium 4.7.2 libraries. Participants were recontacted using the phone numbers provided in the original “SARS-CoV-2 epidemiological survey in the population of Ribeirão Preto” informed consent form, which authorized storage of biological material and future contact for additional research. In this message, a link to a Google Form was sent, where participants could agree or decline to participate in the study. If they did, they would confirm their personal information for identification (name, national ID, gender, age, birthdate, and address), complete the Leprosy Suspicion Questionnaire (LSQ), authorization to access the stored material (serum) and acceptance of the suspected individual [specific markings of signs and symptoms in the LSQ, associated with positivity in APGL-I and anti-Mce1A serological tests] for a neurodermatological clinical evaluation by dermatologists and leprologists (Fig. 1). All sociodemographic and clinical data were accessed using coded identifiers to ensure confidentiality.

### Leprosy suspicion questionnaire (LSQ) and MaLeSQs^®^

The digital LSQ consists of 14 questions that investigate neurodermatological signs and symptoms of leprosy (Table 1). The questions must be answered with “Yes” or “No”. Positivity is assessed by any question marked for screening the diagnosis of leprosy [28]. The registered questionnaire can be downloaded at https://www.crndsh.com.br/qsh both in Portuguese and English.

**Table 1:** The 14 questions of the registered leprosy suspicion questionnaire

Id	Question
q1	Do you feel numbness in your hands and/or feet?
q2	Tingling (pricking)?
q3	Anesthetized areas in the skin?
q4	Muscle cramps?
q5	Stinging sensation?
q6	Spots on the skin? (do not consider those from birth)
q7	Pain in the nerves?
q8	Nodules on the skin?
q9	Swelling of hands and feet?
q10	Swelling of face?
q11	Weakness in hands? Hard to button shirt? Wear glasses? Write? Hold pans?
q12	Weakness in feet? Difficulty wearing sandals, slippers?
q13	Loss of eyelashes? Loss of eyebrows?
q14	Does anyone in your family have or have had leprosy?

Id: identification. q: questions

To increase the effectiveness, performance, and agility of analysis and prediction in clinical screening diagnosis using LSQ, an artificial intelligence screening model called Machine Learning for Leprosy Suspicion Questionnaire and Screening (MaLeSQs^®^) was applied [28]. The model receives an LSQ as input and outputs a positive or Negative Value depending on the fill pattern. This output is based on all the knowledge acquired by learning patterns from previously filled LSQ where patients were also evaluated by leprologists and given or not the diagnosis for the disease. So, this is not just an analysis of questions individually, but of the combinations of signs and symptoms with the most significant association and accuracy [28]. The responses (*n* = 224) were obtained with LSQ and applied in MaLeSQs^®^.

### Serological analysis

The IgA, IgM, and IgG anti-Mce1A and IgM anti-PGL-I antibodies in 195 serum samples from patients who consented to the use of their stored biological material were measured by indirect ELISA. The recombinant Mce1A protein and PGL-I assay [natural-octyl disaccharide conjugated to bovine serum albumin (ND-O-BSA; NR-19346; BEI Resources)] was applied as described by Lima et al. (2023). The assay results were recorded as the mean optical density (O.D.) of the samples in triplicate, after subtraction of the blank control. The assay was repeated if the coefficient of variation was > 10%. The index was calculated by dividing the mean O.D. by the established cut-off, and values ​​greater than 1.0 were considered positive. The results in the final report were classified as follows: <1.0, Negative; >1.0, Positive; >1.0 ≤ 1.5, Low risk; >1.5 ≤ 2.0, Moderate risk; > 2.0, High risk [21, 22, 29].

### Molecular diagnosis (qPCR-RLEP)

The assay for DNA identification of *M. leprae* was performed according to the protocol by Azevedo et al. (2017) and further described by Lima et al. (2022, 2023) in dermal scrapings allocated on filter paper collected from four sites (both earlobes, elbow, knee, and/or skin lesion). Detection of *M. leprae* was performed by real-time polymerase chain reaction (qPCR) for the *M. leprae*-specific repetitive element (RLEP) target located throughout the bacillus genome, a cycle threshold (Ct) value > 45 was considered undetectable. All reactions included a control without DNA and a positive control with DNA extracted from a biopsy of a patient with lepromatous leprosy. Molecular diagnosis was used as a qualitative test to detect the presence of the bacillus and as a quantitative test to assess the bacillary load in the evaluated suspected cases [23, 30].

### Clinical diagnosis

Individuals (*n* = 37) who agreed to undergo clinical evaluation were examined according to the case definition criteria described below. Case definition: Individuals underwent a standardized neurodermatological clinical examination according to the guidelines of the Brazilian Ministry of Health and the WHO guidelines, using the recommended cardinal signs [31]. The diagnosis of leprosy was made upon detection of at least one of the following signs/symptoms: (a) lesion(s) and/or area(s) of the skin with altered thermal and/or pain and/or tactile sensitivity; (b) thickening of the peripheral nerve(s), associated with sensory and/or motor and/or autonomic alterations; and/or (c) presence of *M. leprae*, confirmed by slit-skin smear bacilloscopy or skin biopsy [32, 33], which for this work was confirmed by RLEP-qPCR [30]. Auxiliary tests for clinical diagnosis were used, including assessment of tactile sensation with a Semmes-Weinstein esthesometer, as well as complementary exams such as serology and molecular exams [23]. If positive, the respective patients were referred to the basic health unit closest to their residence or municipal reference center to receive specific treatment with MDT.

### Statistical analysis

The data were analyzed by GraphPad Prism v. 9.0 software (GraphPad Inc., La Jolla, CA, United States). The chi-squared test was used to assess associations among categorical variables and the positivity of exams. Study population characteristics were analyzed by a t-test and chi-squared test. The Kruskal–Wallis test followed by Dunn’s test, and Mann-Whitney test was used to analyze antibody level variations. The level of statistical significance was set by the alpha value (5%). The level of statistical difference was set at *P* < 0.05. Venn diagrams were generated using the online tool Draw Venn Diagram to represent the overlap in the number of positive exams (http://bioinformatics.psb.ugent.be/webtools/Venn/). To compare the performance of the different serological tests and their combination with MaLeSQs^®^ in clinically evaluated and diagnosed individuals, sensitivity, specificity, positive predictive value (PPV), and negative predictive value (NPV) were calculated using Microsoft Excel 365 v. 16.43.

To perform georeferencing, the residential addresses were remotely geocoded using BatchGeo Pro (//batchgeo.com), and the QGIS 3.28.13-Firenze software (http://www.qgis.org) was used to produce the distribution maps of individuals in the municipality. The mapping was classified as geoepidemiology with antibodies and was categorized into scales for evaluating negative serologies (< 1.0), the degree of positivity (> 1.0), and the severity of the indices.

All spatial analyses were conducted at the census tract level for the municipality of Ribeirão Preto (*n* = 1,431 census tracts), which served as the areal unit of analysis. Global spatial autocorrelation analysis was performed using the univariate Moran’s I index [34] applied to the serological scores for IgM anti-Mce1A, antibody associated with active disease or potential subclinical infection [22], and the number of leprosy cases aggregated by census tract. To identify spatial association patterns between two variables, the Local Bivariate Moran’s I spatial autocorrelation statistic was applied using QGIS software and GeoDa software (version 1.20.0.20). This analysis allowed for the detection of significant spatial clusters between the analyzed variables, based on spatial weights defined by contiguity-based neighborhood structures (Supplementary material). The spatial weight matrix used was first-order contiguity (Queen), and 9,999 permutations were performed for significance testing. To assess the presence of spatial correlation between the distribution of diagnosed cases, IgM anti-Mce1A seropositivity, and sociodemographic factors in the city of Ribeirão Preto, a set of indicators stratified by census tract was selected, including: (a) average number of residents per household; (b) per capita household income; (c) proportion of households with access to piped water; (d) proportion of households with basic sanitation; (e) proportion of households with access to electricity; and (f) census tract classification according to the São Paulo State Social Vulnerability Index (IPVS). Local spatial autocorrelation analysis was performed using univariate Local Indicators of Spatial Association (LISA) statistics in GeoDa software to identify the presence and location of significant spatial clusters. Adjustment for multiple testing was performed using the False Discovery Rate (FDR) method. The bivariate LISA statistic was applied to the relationship between the number of leprosy cases and the selected sociodemographic variables listed, and the analysis was performed using a spatial contiguity weight matrix (Queen), with 999 permutations. FDR correction was applied to control for multiple tests (Supplementary material).

Spatial weights were defined using a first-order Queen contiguity matrix based on shared borders and vertices. The connectivity histogram was inspected to ensure no census tracts were isolated (islands); the minimum number of neighbors was 1, and the mean was 6.11. To ensure the reliability of the statistical inference and mitigate potential instability from boundary effects, significance testing for both Global and Local Moran’s I was conducted using a conditional permutation approach (Monte Carlo randomization) with 999 permutations. The significance level was set at *P* < 0.05, adjusting for multiple comparisons in local analyses using the FDR method.

## Results

### Diagnostic screening responses with LSQ and MaLeSQs^®^

**Fig. 2:**
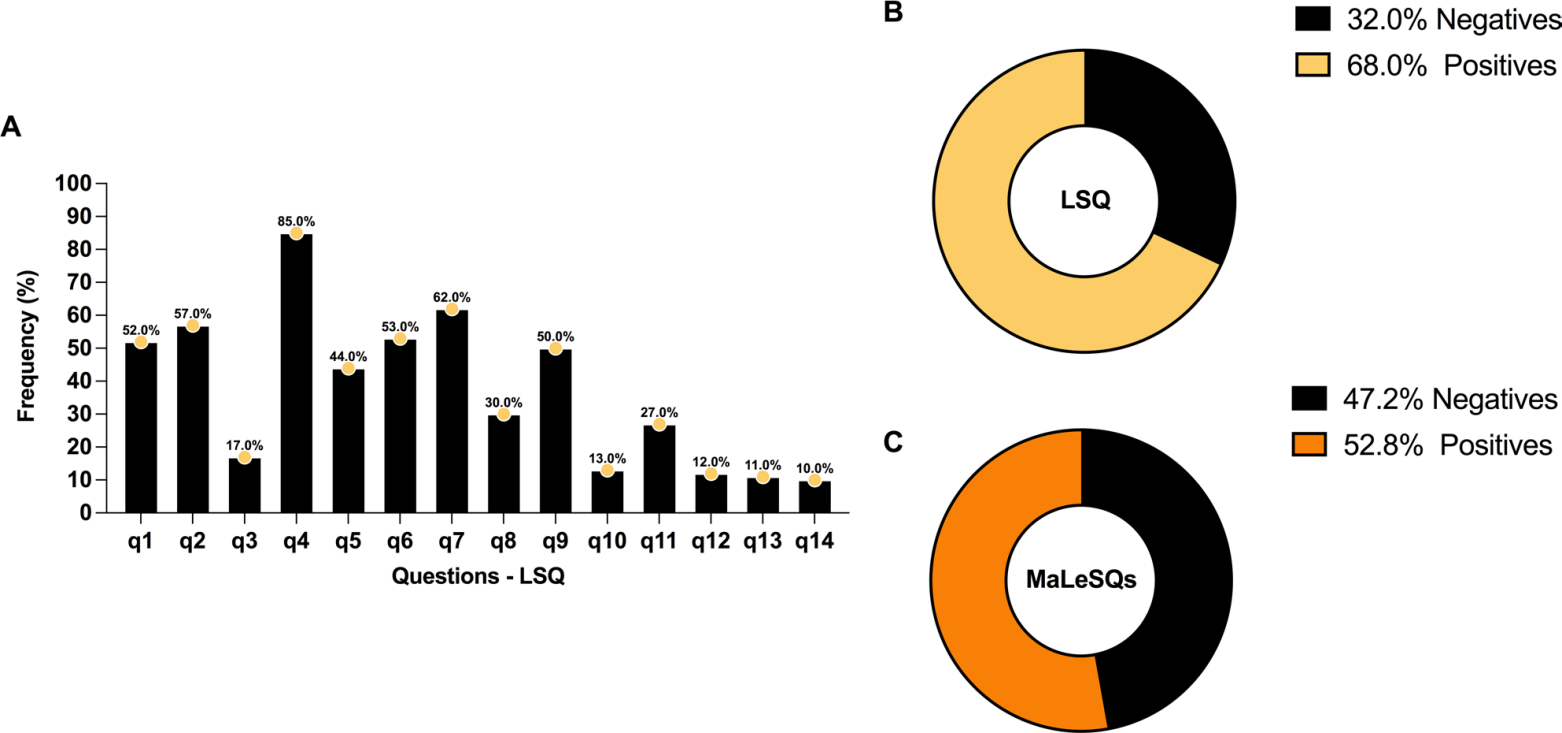
Frequency of individuals according to signs and symptoms of leprosy identified in the diagnostic screening. Frequency of questions marked on the LSQ (**A**-**B**) and MaLeSQs^®^ (**C**)

The number of questionnaires answered was 224 and 72 (32.1%) individuals did not answer any questions. The assessment highlights the predominance of neurological symptoms through the positive responses to the most frequently asked questions, specifically question 4 (q4): Do you feel numbness in your hands and/or feet?; q7: Tingling (pricking)?; q2: Muscle cramps?; q1: Pain in the nerves?; q9: Swelling of hands and feet?. With q6 (Spots on the skin?) being only the fifth most frequently answered (Fig. 2A).

Using traditional LSQ, the analysis performance identified 11 true positives, 19 false positives, 1 false negative, and 9 true negatives. Resulting in a sensitivity of 91.7%, specificity of 32.1% and 68% positivity (Fig. 2B). Using artificial intelligence by MaLeSQs^®^, 10 true positives, 15 false positives, 2 false negatives, and 13 true negatives were obtained. Being performed with a sensitivity of 83.3%, specificity of 46.4% and positivity of 52.8% (Fig. 2C). Without MaLeSQs^®^, 30 people would be evaluated for 11 diagnoses, while with MaLeSQs^®^, 25 people would be assessed for 10 diagnoses. This represents a decrease of 9.1% in sensitivity, but an increase of 44.4% in specificity.

### Case detection

One hundred ninety-five (*n* = 195) individuals agreed to an appointment for neurodermatological evaluation, and only 37 individuals (18.9%) attended it, which were conducted over three attempts in different months of 2021-2022-2023. Twelve (*n* = 12) new cases were diagnosed (32.4%). Considering only the new cases reported in the study, the detection rate of new cases in the general population of the municipality was 1.69/100,000 inhabitants and 6.15% in the population sample evaluated in the study.

Among the general study population, 3.3% had a family history of leprosy, and 8.2% were declared as contacts of patients with the disease. The diagnoses performed demonstrate the characteristic of being a contact (7/12; 58.3%) and having a family history of leprosy (2/12; 16.7%), higher than in the general population (*P* < 0.0001). The diagnosed patients were classified as having the MB form (100%) and a borderline clinical form (100%), with a predominance of neurological symptoms over dermatological signs. Among the new cases, 58.3% (7/12) were diagnosed with physical disability grade 1 (G1D), characterized by decreased tactile sensation in the hands and/or feet, with sensitivity only for stimuli greater than 2 g-force (gf) (Table 2).

**Table 2:** Description of demographic and clinical data of individuals (*n* = 195)

	Geral *n* = 183	New Cases *n* = 12	*P* value
**Age**,** years**,** median (IQR)**	54.0 (41–67)	54.0 (48–64)	0.58^a^
**Sex**,** n (%)**			0.94^b^
Male	78 (42.6)	5 (41.7)	
Female	105 (57.4)	7 (58.3)	
**Operational Classification**,** n (%)**			-
PB	-	0 (0)	
MB	-	12 (100)	
**Clinical Form**,** n (%)**			-
Borderline	-	12 (100)	
**Physical disability**,** n (%)**			-
Grade 0	-	5 (41.7)	
Grade 1	-	7 (58.3)	
Grade 2	-	0 (0)	
**qPCR-RLEP**,** n (%)**			-
Positive	-	0 (0)	
Negative	-	12 (100)	
**Household contact (HHC)**,** n (%)**			**< 0.0001** ^**b**^
Yes	15 (8.2)	7 (58.3)	
No	168 (91.8)	5 (41.7)	
**Family History of Leprosy**,** n (%)**			**< 0.0001** ^**b**^
Yes	6 (3.3)	2 (16.7)	
No	177 (96.7)	10 (83.3)	

IQR, interquartile range; PB, paucibacillary; MB, multibacillary. (a) Mann-Whitney test. (b) Chi-square test

### Anti-Mce1A and APGL-I serology as biomarkers

**Fig. 3:**
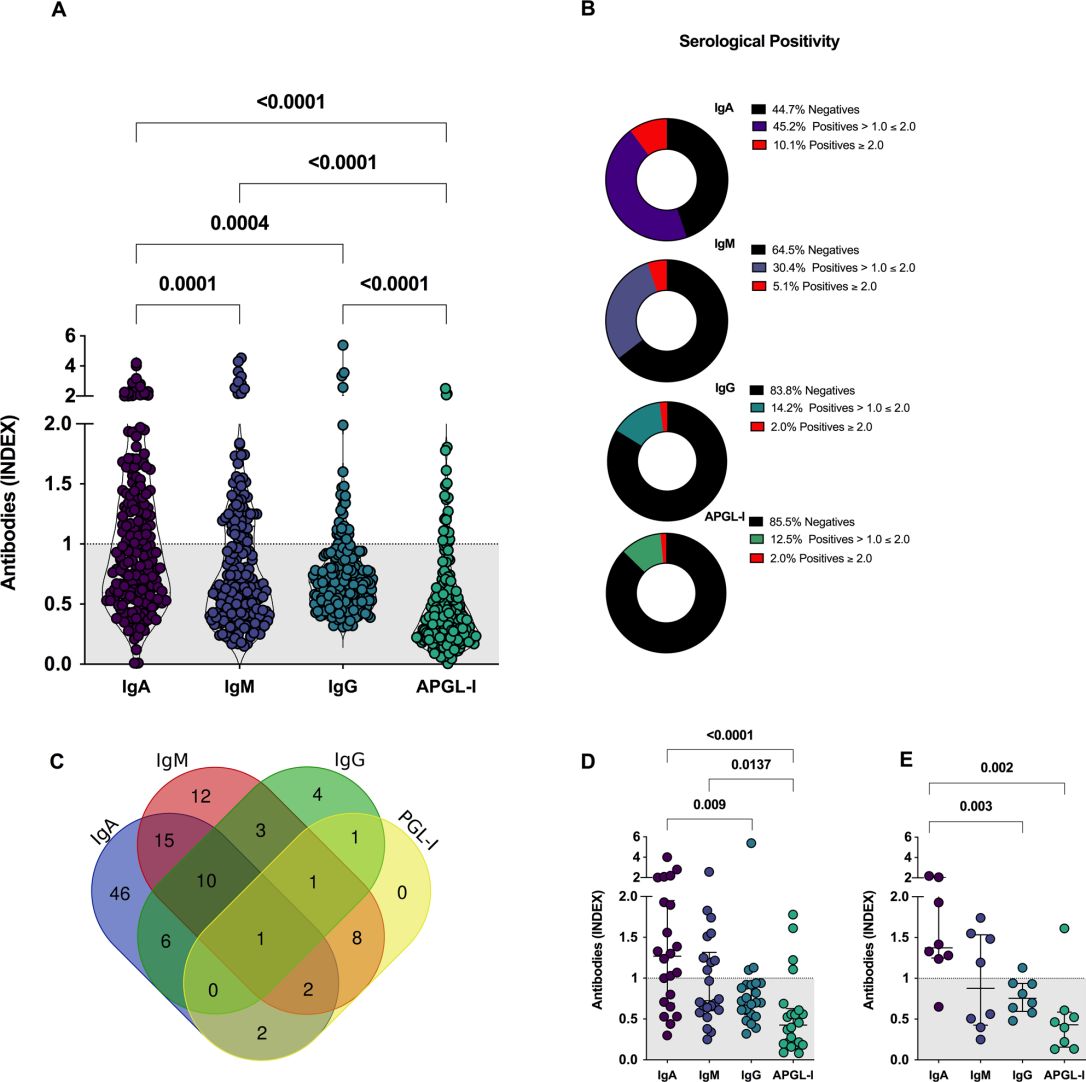
Performance of antibodies as biomarkers of leprosy. Evaluation of the levels of IgA, IgM, and IgG anti-Mce1A and IgM anti-PGL-I (APGL-I) antibodies in serum samples (*N* = 195). Statistical difference was assessed by the Mann-Whitney test and Kruskal-Wallis test (**A**). Panel of the frequency of serological results (**B**). Overlap of positive results for the antibodies tested (**C**). Levels of antibodies in serum samples from contacts of leprosy patients (**D**). Levels of antibodies in serum samples from individuals with a history of leprosy in the family (**E**). The indices were calculated by dividing the optical density (O.D. 450 nm) of each sample by the cut-off. Indices above 1.0 were considered positive

Among serum samples analyzed (*n* = 195), the IgA anti-Mce1A ELISA showed the highest seropositivity (55.3%), the highest rates [median = 0.93 (IQR = 0.59–1.41)] and a significant difference as compared to the rates of the IgM ELISA [seropositivity = 35.5.%; median = 0.65 (IQR = 0.41–1.14); *P* = 0.0001], IgG [seropositivity = 16.2%; median = 0.68 (IQR = 0.52–0.88); *P* = 0.0004] and IgM APGL-I [seropositivity = 14.5%; median = 0.37 (IQR = 0.22–0.60); *P* < 0.0001]. The IgM and IgG anti-Mce1A serology showed higher rates (*P* < 0.0001) as compared to the APGL-I serology. Indices with values ​​greater than or equal to 2.0 were more frequently detected in IgA (10.1%) and IgM (5.1%) anti-Mce1A serologies (Fig. 3A-B). The overlap of positivity with the antibodies tested highlights the greater involvement of double or triple positives when Mce1A serology was used. As well as the lower involvement of PGL-I serology when performed in parallel with Mce1A serology (Fig. 3C). Individuals classified as contact of leprosy patients presented IgA [median = 1.26 (IQR = 0.69–1.95); *P* < 0.0001] and IgM [median = 0.72 (IQR = 0.60–1.31); *P* = 0.013] levels higher than APGL-I [median = 0.42 (IQR = 0.19–0.62);]. IgG anti-Mce1A showed levels with a median of 0.71 (IQR = 0.55–0.92) and lower rates compared to IgA (*P* = 0.009) (Fig. 3D). The analysis of volunteers with a history of leprosy in the family demonstrated IgA anti-Mce1A [median = 1.37 (IQR = 1.24–2.02)] higher levels than IgG [median = 0.75 (IQR = 0.59–0.93); *P* = 0.003], and APGL-I [median = 0.43 (IQR = 0.15–0.58); *P* = 0.0024]. IgM anti-Mce1A showed indices with a median of 0.87 (IQR = 0.42–1.53) (Fig. 3E).

### Serological assay for new cases detected

**Fig. 4:**
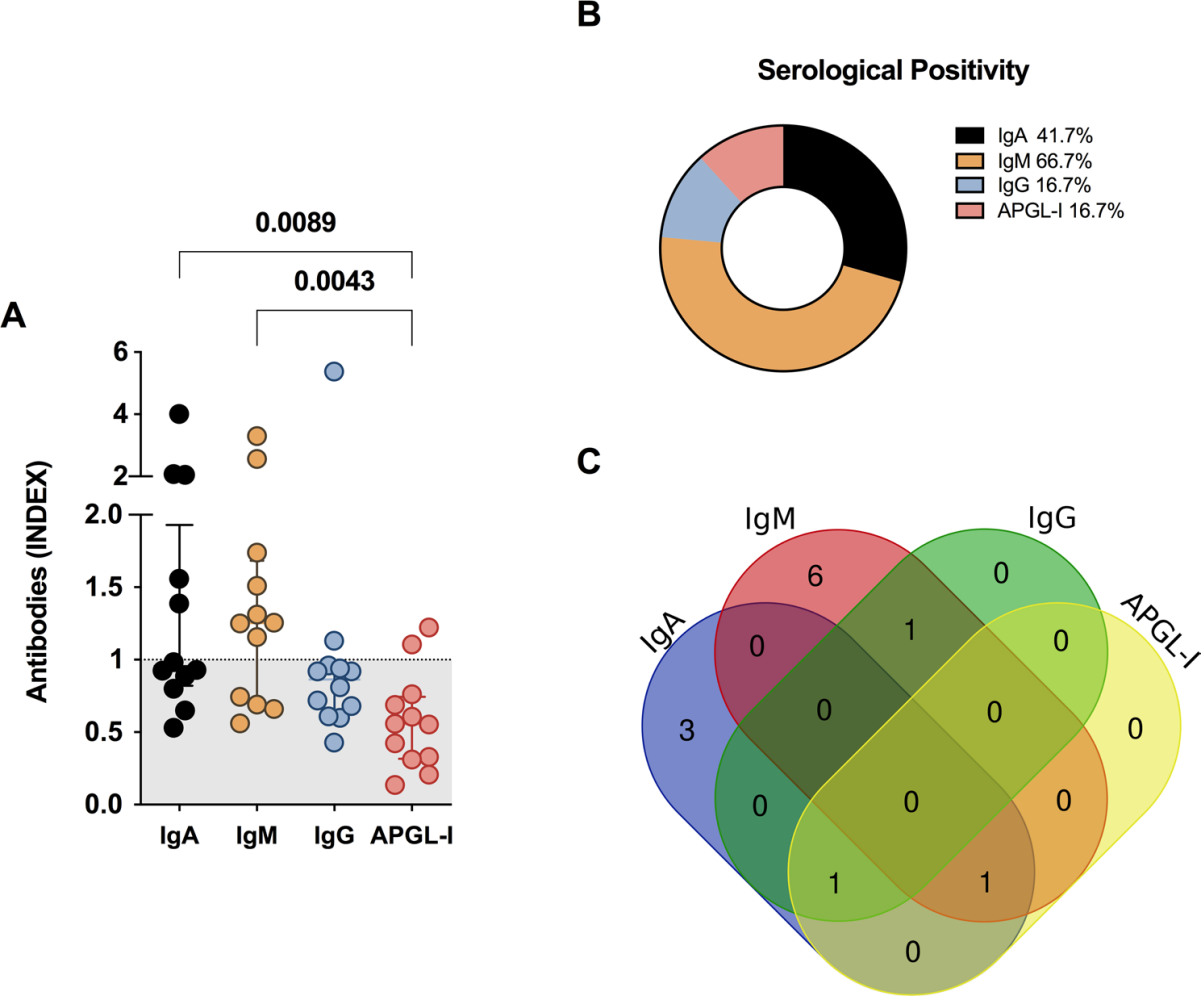
Performance of antibodies as biomarkers of leprosy in newly diagnosed cases. Assessment of the levels of IgA, IgM, and IgG anti-Mce1A and IgM anti-PGL-I antibodies in serum samples (*n* = 12) and Statistical difference assessed by the Mann-Whitney test and Kruskal-Wallis test (**A**). Panel of the frequency of serological results (**B**). Overlay of positive results for the antibodies tested (**C**). The indices were calculated by dividing the optical density (O.D. 450 nm) of each sample by the cut-off. Indices above 1.0 were considered positive

IgM anti-Mce1A serology was positive in 66.7% [8/12 cases; median = 1.25 (IQR = 0.70–1.68)] of new cases and in parallel with IgM serology that diagnosed 41.7% [5/12 cases; median = 0.95 (IQR = 0.82–1.93). IgG anti-Mce1A [median = 0.86; IQR = 0.62–0.95] and IgM APGL-I [median = 0.55; IQR = 0.31–0.74] serologies showed the lowest seropositivity in patients. ELISA IgA (*P* = 0.008) and IgM (*P* = 0.004) showed a statistical difference as compared with APGL-I serology (Fig. 4A-B). The overlap of positive results demonstrates a higher frequency of results with the inclusion of IgM anti-Mce1A serology, and the low potential of anti-Mce1A IgG and anti-PGL-I IgM for detecting diagnosed cases (Fig. 4C).

### Performance of screening tools in clinically evaluated and detected cases

Analyses were performed for the serological marker’s IgA, IgM, IgG anti-Mce1A, and IgM anti-PGL-I. For IgM anti-Mce1A showed the best diagnostic performance, with a sensitivity of 66.7%, specificity of 40.0%, PPV of 44.4%, and NPV of 78.9%. The results for the other markers can be found in Table 3.

**Table 3:** Diagnostic performance of serological biomarkers

	Sensitivity	Specificity	PPV	NPV
IgA anti-Mce1A	41.7%	36.0%	35.7%	69.6%
IgM anti-Mce1A	66.7%	40.0%	44.4%	78.9%
IgG anti-Mce1A	16.7%	11.5%	40.0%	69.7%
IgM anti-PGL-I	16.7%	8.0%	50.0%	69.7%

PPV, Positive predictive value; NPV, negative predictive value

To further evaluate the diagnostic potential of combining serology with artificial intelligence, performance metrics were calculated for the integration of IgM anti-Mce1A with MaLeSQs^®^. Two different approaches were considered (A) IgM anti-Mce1A + MaLeSQs (AND condition): a case was classified as positive only when both IgM and MaLeSQs^®^ were positive; (B) IgM anti-Mce1A + MaLeSQs (OR condition): a case was classified as positive when at least one of the two tests was positive. As showed in Table 4, the AND condition resulted in a sensitivity of 50.0%, specificity of 24.0%, PPV of 50.0%, and NPV of 76.0%. In contrast, the OR condition achieved markedly higher sensitivity (100%) and NPV (100%), while also improving specificity to 68.0%, although the PPV remained at 41.4%.

**Table 4:** Diagnostic performance of IgM anti-Mce1A combined with MaLeSQs^®^ under AND and OR conditions

	Sensitivity	Specificity	PPV	NPV
IgM + MaLeSQs (AND)	50.0%	24.0%	50.0%	76.0%
IgM + MaLeSQs (OR)	100%	68.0%	41.4%	100%

PPV, Positive predictive value; NPV, negative predictive value

### Analysis of seropositivity and signs and symptoms of LSQ

Initially, the antibody levels of each test were compared with those of LSQ-positive (LSQ+) and LSQ-negative (LSQ-) individuals. LSQ + individuals presented higher IgA anti-Mce1A levels [median = 1.00 (IQR = 0.63–1.63); *P* = 0.016] compared to LSQ- [median = 0.86 (IQR = 0.52–1.17)]. IgM anti-Mce1A did not differ between the LSQ+ [median = 0.64 (IQR = 0.40–1.19); *P* = 0.90] and LSQ- [median = 0.71 (IQR = 0.43–1.05)] groups. Anti-Mce1A IgG serology had higher levels in LSQ + individuals [median = 0.70 (IQR = 0.54–0.98); *P* = 0.030] compared with LSQ- [median = 0.64 (IQR = 0.46–0.80)]. However, APGL-I showed higher levels in LSQ- [median = 0.42 (IQR = 0.28–0.74) *P* = 0.015] compared with LSQ+ [median = 0.35 (IQR = 0.20–0.55)].

**Fig. 5:**
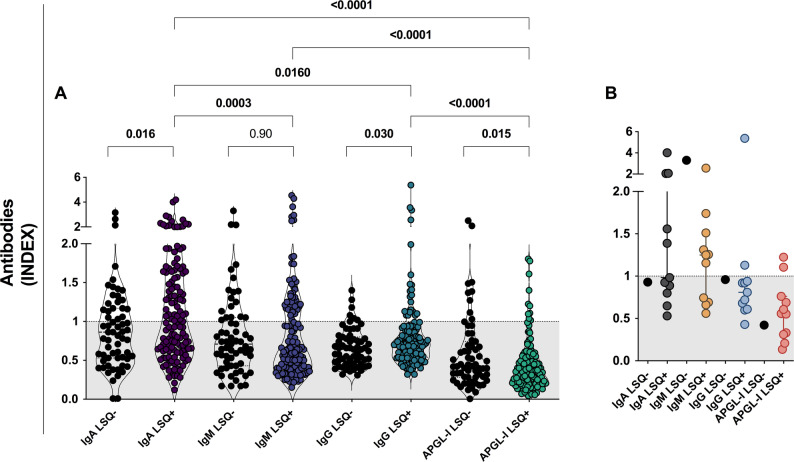
Performance of antibodies as biomarkers of leprosy in relation to LSQ positivity. Evaluation of the levels of IgA, IgM, and IgG anti-Mce1A and IgM anti-PGL-I (APGL-I) antibodies in serum samples (*N* = 195). Statistical difference was assessed by the Mann-Whitney test and Kruskal-Wallis test (**A**-**B**). Levels of IgA, IgM, and IgG anti-Mce1A and IgM anti-PGL-I (APGL-I) antibodies in serum samples of diagnosed patients (**B**)

Differences were observed in the inter-assay indices when the LSQ showed positivity, comparing IgA LSQ + with IgM LSQ+ (*P* = 0.0003), IgG LSQ+ (*P* = 0.01), and APGL-I LSQ+ (*P* < 0.0001). IgM LSQ + and IgG LSQ + were statistically different (*P* < 0.0001), as compared with the APGL-I LSQ + group. The data were analyzed by comparing antibodies of the same class and other classes, as well as within the LSQ + and LSQ- groups (Fig. 5A). Only 8.3% (1/12) of the diagnosed patients had a negative LSQ, while 45.4% (5/11) were IgA LSQ+, 63.6% (7/11) were IgM LSQ+, and IgG LSQ + obtained the same result as APGL-I LSQ+ [18.2% (2/11)] (Fig. 5B).

### Georeferencing with serological biomarkers

**Fig. 6:**
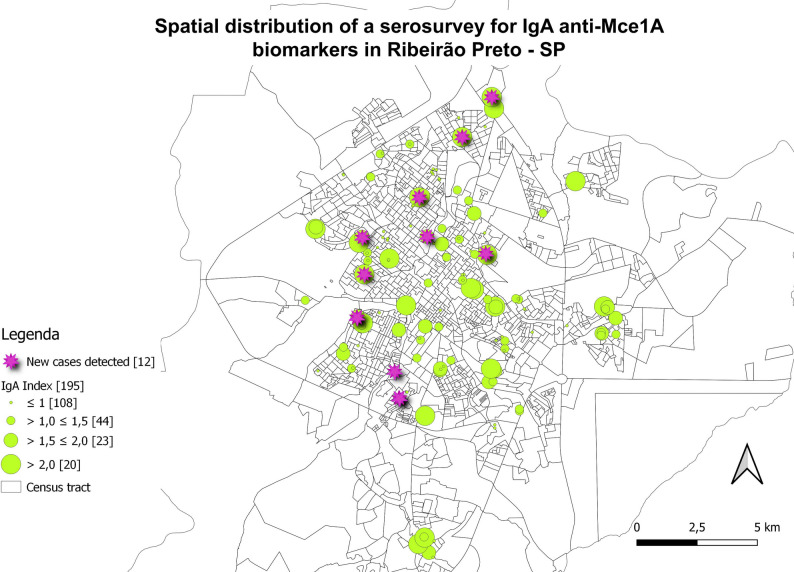
Spatial distribution of serological research for anti-Mce1A biomarkers. IgA ELISA indices for *N* = 195 individuals. Newly diagnosed cases (*n* = 12; 32.4%) of *n* = 37 clinically evaluated patients were highlighted in pink circles. Indices above 1.0 were considered positive, and above 2.0 were of higher risk. ELISA indices were represented in ascending order (green circles), and the number of individuals tested is shown in brackets. Spatial distribution of newly detected leprosy cases; two are household contacts and two reside on the same street, with overlapping addresses resulting in 10 cases shown on the map

**Fig. 7:**
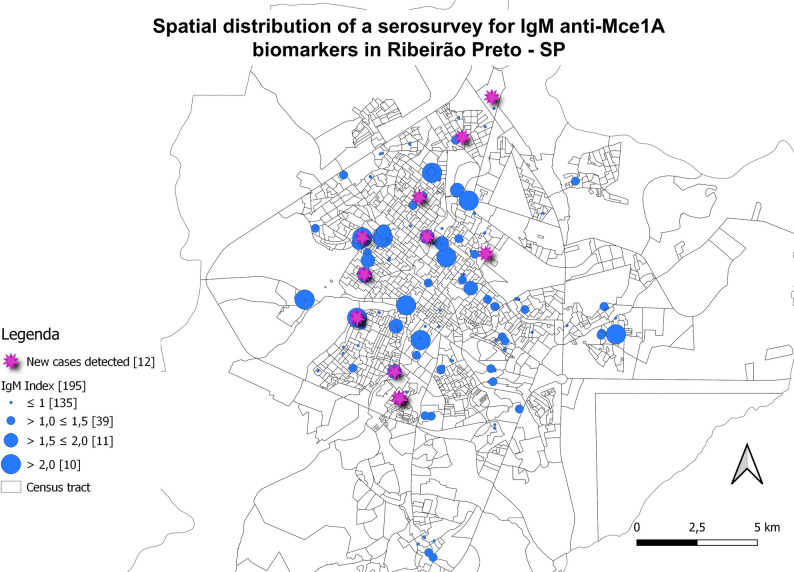
Spatial distribution of serological research for anti-Mce1A biomarkers. IgM ELISA indices for *N* = 195 individuals. Newly diagnosed cases (*n* = 12; 32.4%) of *n* = 37 clinically evaluated patients were highlighted in pink circles. Indices above 1.0 were considered positive, and above 2.0 were of higher risk. ELISA indices were represented in ascending order (blue circles), and the number of individuals tested is shown in brackets. Spatial distribution of newly detected leprosy cases; two are household contacts and two reside on the same street, with overlapping addresses resulting in 10 cases shown on the map

**Fig. 8:**
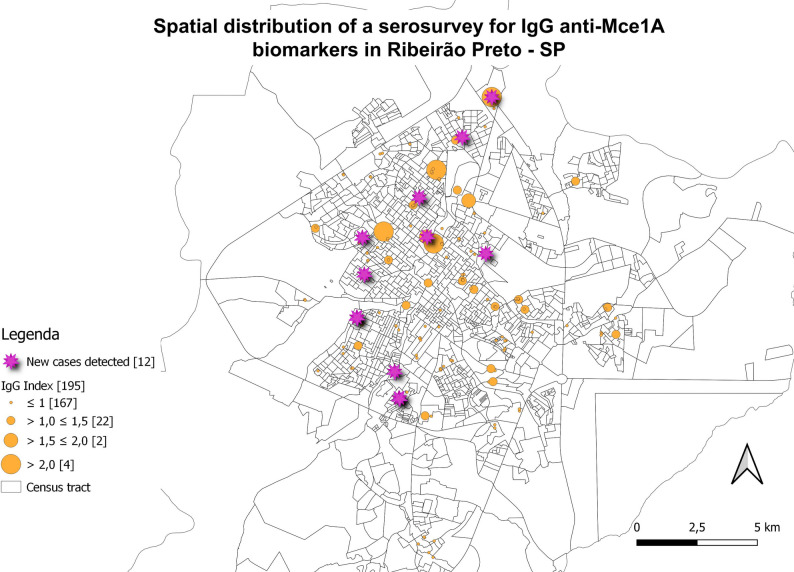
Spatial distribution of serological research for anti-Mce1A biomarkers. IgG ELISA indices for *N* = 195 individuals. Newly diagnosed cases (*n* = 12; 32.4%) of *n* = 37 clinically evaluated patients were highlighted in pink circles. Indices above 1.0 were considered positive, and above 2.0 were of higher risk. ELISA indices were represented in ascending order (orange circles), and the number of individuals tested is shown in brackets. Spatial distribution of newly detected leprosy cases; two are household contacts and two reside on the same street, with overlapping addresses resulting in 10 cases shown on the map

**Fig. 9:**
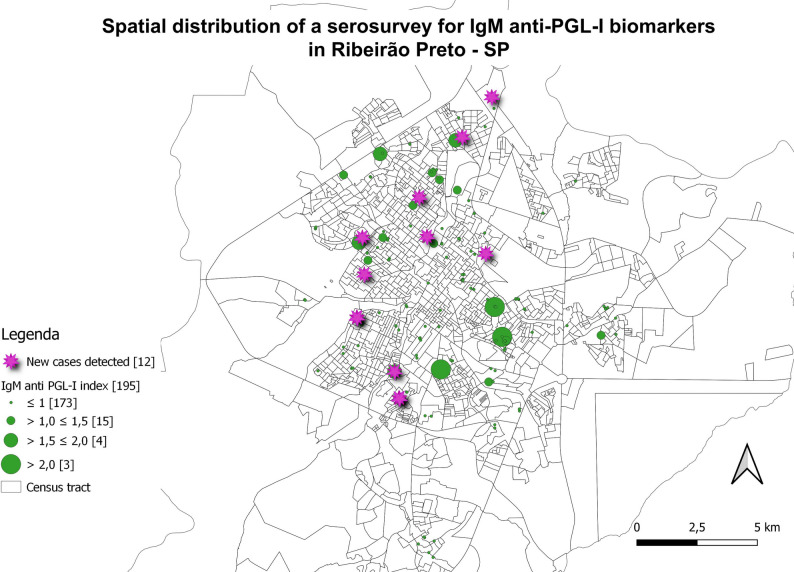
Spatial distribution of serological research for anti-PGL-I biomarkers. IgM ELISA indices for *N* = 195 individuals. Newly diagnosed cases (*n* = 12; 32.4%) of *n* = 37 clinically evaluated patients were highlighted in pink circles. Indices above 1.0 were considered positive, and above 2.0 were of higher risk. ELISA indices were represented in ascending order (green circles), and the number of individuals tested is shown in brackets. Spatial distribution of newly detected leprosy cases; two are household contacts and two reside on the same street, with overlapping addresses resulting in 10 cases shown on the map

New cases diagnosed in the study and individuals with samples tested in serology were marked on maps and associated with the residence locations to assess spatial distribution (Figs. 6, 7, 8 and 9). The evaluation of serology and diagnoses confirms the presence of a distribution throughout the municipality. Serology with the IgA antibody presented the highest rates and served as an alert for contact with the bacillus (Fig. 6).

The assessment of the presence of clusters and spatial correlations was developed based on the seropositivity of the anti-Mce1A IgM antibody, which is the most critical molecule in the diagnosis of confirmed cases. No significant spatial autocorrelation was detected based on the permutation test (*P* > 0.05), indicating the absence of a grouped spatial pattern for serological values ​​in the analyzed territory. In addition, demographic data, such as the average household income of the census sector and the illiteracy rate, were grouped and cross-referenced with the spatial distribution; however, no spatial correlation was found between the cases and these indices. Moran’s I value was close to zero and not significant (*P* > 0.05), indicating the absence of global spatial autocorrelation for leprosy cases and distribution of patients with positive serology for IgM anti-Mce1A in the census tracts analyzed. This suggests a random spatial distribution of these variables in the studied territory, with no evidence of global clusters of high or low incidence (Supplementary Material – Figures S1-S4).

The LISA cluster map (Supplementary Material – Figures S5-S6) demonstrated the absence of relevant spatial clusters for leprosy cases and serology, reinforcing the Moran Global findings regarding the spatial randomness of the disease distribution in the analyzed territory. Thus, no pair of variables showed a statistical difference in local spatial association (Supplementary Material – Table S1).

## Discussion

Actively searching for new cases and examining household contacts are the primary strategies for controlling leprosy [3, 35], as well as increasing the identification of latent leprosy cases in low-endemic areas [36]. The study utilized samples originally collected for the investigation of SARS-CoV-2 infection and given the survey’s scope and representativeness of the municipality, these stored specimens and sociodemographic data served solely as the basis for conducting the seroepidemiological evaluation of leprosy biomarkers and their spatial distribution. The use of clinical screening through questionnaires and serology tests ensured the recruitment of priority cases, serving as a strategy for characterizing exposure to the bacillus and active disease in the municipality. Early detection through contact tracing and active surveillance is essential to break the chain of transmission [37].

The analysis of the epidemiological history of the individuals revealed that 58.3% (7/12) of the diagnosed cases were contacts of leprosy patients, and 16.7% (2/12) reported a family history of the disease. Individuals not clinically evaluated demonstrate a higher prevalence of contact with the bacillus, as well as a family history in 8.2% and 3.3%, respectively. Contacts of leprosy patients exhibit a high risk of developing leprosy, and contact tracing is helpful for early diagnosis [38]. The risk of living with a primary case of leprosy may override individual and geographic risk factors for becoming a leprosy case [39]. The clinical evaluation of the cases highlights the diagnosis of MB cases (100%) and the presence of physical disability at diagnosis (G1D) in 58.3% (7/12) of cases. Higher leprosy rates among contacts of patients with MB leprosy may be explained by exposure to a relatively higher bacillary load [39]. The use of an artificial intelligence tool, such as MaLeSQs^®^, is an interesting approach to improve performance, accelerating early detection, reducing the need for clinical evaluation of a larger number of individuals at low risk for the disease, increasing screening specificity, especially in large populations [28].

Serological biomarkers can help reduce transmission by facilitating the early identification of leprosy cases and enabling contact screening. The host immune responses involving immune polarization pose an essential challenge for detection across the clinical spectrum of the disease, including subclinical infections and oligosymptomatic cases. Thus, the performance of these antibodies in identifying potentially infected contacts and predicting the development of the disease is a long-term goal [40]. This way, elimination with early diagnosis will not be possible without these new tools and technologies in the context of neglected tropical diseases [41]. The study employed the most widely applied APGL-I antibody detection method, as well as the use of other markers against the Mce1A protein, to expand case-tracking tools.

Goulart et al. (2008) demonstrated that evaluating APGL-I humoral immunity identified 39.3% of positive results among contacts with progression to active disease, compared to a positivity rate of 10.4% among healthy contacts, representing a relative risk almost six times higher for disease development in the positive cases. Contacts of LL patients presented a 3.8-fold-higher risk of developing leprosy [10]. Indices to IgM anti-PGL-I over 2.0, which is comparable to MB cases, exhibiting a high bacillary index, indicating a higher risk of leprosy in the future [29]. Lima et al. (2022) demonstrated in a population study from northeast Brazil that individuals with anti-Mce1A IgM-positive antibodies had a 3.6 times higher chance of being diagnosed with leprosy, while those with IgA-positive antibodies had a 2.3 times higher chance. The subsequent study with patients from southeast Brazil (Ribeirão Preto city) confirmed the association and clustering with anti-Mce1A biomarkers, reinforcing IgA as a signature of contact with the bacillus, IgM with better performance for diagnosing new cases and active disease, and IgG as a marker of prolonged contact and monitoring of cases in treatment [22].

The use of serological diagnosis enables the tracking of household contacts with a higher risk of developing active disease, contributing to the monitoring of cases and serving as a tool for predicting leprosy reactions and identifying the risk of relapse [42]. Serological analysis anti-Mce1A reveals high seropositivity in the evaluated individuals, with 55.3% for IgA and 35.5% for IgM, indicating a higher risk result (> 2.0) in 10.1% of the samples tested for IgA and 5.1% for IgM. The seropositivity rates for IgG anti-Mce1A and IgM anti-PGL-I were similar, at 16.2% and 14.5%, respectively. The data support the presence of *M. leprae* circulation, as indicated by serological evaluation using two different antigens, albeit in a smaller proportion of high-risk cases. The comparison of IgM antibodies against distinct antigens reinforces the greater sensitivity and accuracy of the anti-Mce1A ELISA, which detected 66.7% of cases, compared to the APGL-I ELISA, which had a detection capacity of 16.7% in the study patients. IgA serology was positive in 41.7% of the cases evaluated and has superior potential for confirming cases. However, follow-up studies of seropositive individuals are still needed to verify biomarkers as predictors of progression to active disease and/or detection of subclinical infection. The lack of tools for detecting cases in all forms of leprosy, particularly before the appearance of signs and symptoms, is a challenge and a limitation in the laboratory diagnosis of the disease [43].

In the present study, 87.5% (7/8) of individuals with a family history of leprosy were positive for the IgA anti-Mce1A test, and 50% (4/8) were positive for IgM, with only 12.5% (1/8) being positive for IgG anti-Mce1A and APGL-I. Among contacts, 54.5% (12/22), 40.9% (9/22), 9.1% (2/22), and 13.6% (3/22) were seropositive for IgA, IgM, and IgG anti-Mce1A and APGL-I, respectively. Interestingly, the anti-Mce1A IgA and IgG biomarkers showed higher levels in individuals with signs and symptoms identified in the clinical screening questionnaire (LSQ). These parameters can be assessed during the screening and clinical investigation of patients, as well as in identifying priority groups for clinical follow-up. The identification of higher IgA levels in questionnaire-positive individuals compared to the other immunoglobulins tested demonstrates their role in assessing exposure to the genus *Mycobacterium*. The low sensitivity and accuracy of APGL-I antibodies were also evident in the higher seropositivity among individuals without signs or symptoms, as identified in the clinical screening questionnaire. As well, a higher seropositivity of all anti-Mce1A antibodies among questionnaire-positive individuals than APGL-I levels.

IgM anti-Mce1A showed the best diagnostic performance as a serological screening tool, with a sensitivity of 66.7% and a specificity of 40.0%. When IgM results were combined with the machine-learning system, sensitivity and specificity were 50.0% and 24.0% under the AND condition, whereas the OR condition (IgM or MaLeSQs^®^ positive) achieved higher sensitivity (100%) and NPV (100%), with a specificity of 68.0%. This comparison demonstrates that different strategies for combining IgM with MaLeSQs^®^ substantially modify diagnostic performance, with the OR condition maximizing sensitivity and ensuring that no cases were missed, while the AND condition produced a more restrictive yet less efficient classification. In this study, the specificity gain reduced unnecessary evaluations and clinical workload without compromising case detection, highlighting this approach as a useful strategy to optimize screening and clinical assessment efforts.

The ecological analysis by Ramos et al. (2022) revealed a clustering pattern of cases in the northern region of the municipality of Ribeirão Preto, characterized by a population with lower income levels and educational attainment [44]. Other studies in other municipalities also showed a higher prevalence of cases in areas of greater social vulnerability [45, 46]. Unlike these studies, our article did not reveal global spatial autocorrelation in the Moran’s I analyses when examining the variables, suggesting that positive serological values are distributed in a dispersed manner and do not present significant clusters in the urban space of the municipality. This finding is relevant, as it may reflect a diffuse transmission pattern of leprosy, with no evident concentration in areas previously known as hyperendemic.

The sampling characteristics of our study, based on antibody testing in a randomized population, prompt us to consider the possibility of silent transmission of leprosy in the municipality, reinforcing the importance of sensitive surveillance strategies throughout the territory, regardless of historically prioritized areas. It should be noted that the studies cited are ecological studies based on notification data; this methodology should be criticized due to its inability to detect the real number of cases in a region, as studies have demonstrated a hidden endemic in many areas of the country as south [47], southeast (region of Ribeirão Preto) [48, 49], and north [50]. In addition, the lack of spatial grouping may also be associated with the relatively small number of new and seropositive cases in the sample universe.

The analysis of the performance of in vitro technologies available to aid in the diagnosis of leprosy allows us to conclude that no laboratory test is capable of detecting all clinical forms of leprosy, and only infected individuals [51]. Even today, diagnosis is mainly based on clinical dermatological and neurological examinations, posing a significant diagnostic challenge [52]. The skills and competencies required for diagnosis, treatment, and management by health professionals are unsatisfactory, leading to late diagnoses, underdiagnosis, physical disabilities, progression to disabilities, socioeconomic impairment, and continued transmission of the *M. leprae*, especially among individuals with low bacillary loads in which leprosy recognition is more challenging [53, 54]. Furthermore, individuals with early leprosy often have low leprosy awareness and disregard the symptoms [53].

Specific antibodies are produced within days or weeks after initial exposure, and the heterogeneity of antibody responses toward protein, carbohydrate, and glycolipid antigens within a clinical group is notable [14, 55]. The satisfactory production of antibodies and their detectability in immunoassays depend on several factors, such as bacillary load, type of immune response, presentation of leprosy, age, nutritional status, severity of the disease, and certain medications or infections, such as human immunodeficiency virus (HIV), that suppress the immune response [56]. The varying endemicity of *M. leprae* and the identification of target analytes with robust performance across leprosy phenotypes complicate the accuracy of tests [57]. However, a negative result does not rule out the clinical diagnosis of leprosy and does not necessarily classify the patient as PB or mild case [32, 57]. The study by Frade et al. (2017) in central-western Brazil showed that 33.1% of healthy individuals (contacts and non-contacts of leprosy) were positive for APGL-I serology and 26.3% for the test using the LID-1 antigen, being considered tests for screening potential cases and with the need for clinical correlation [58]. The study was also developed in a municipality of inner São Paulo state (Brazil), approximately 25 km from the city of Ribeirão Preto. It showed seropositivity for APGL-I in 23.3% of individuals, and 25% of newly diagnosed leprosy cases were positive, as well as one hidden case of leprosy in this supposedly low-endemicity area [29].

Therefore, given the need for a simple, low-cost, and even more sensitive and accurate tool, new technological diagnostic platforms to assess household transmission in less complex health units and a decentralized manner need to be incorporated into the flow of active search and care for leprosy. The recognition of Mce1A protein by unknown host factors is crucial for the internalization of *M. leprae* [59]. Thus, the effectiveness of ELISA for detecting IgA, IgM, and IgG antibodies against the Mce1A protein applied in the north and southeast of the country demonstrated its innovative potential as a test for diagnosing and monitoring leprosy and assessing the level of exposure to the bacillus in the community based on the endemicity of the region [21, 22]. Silva et al. (2021) demonstrated that household contacts exhibit higher IgA seropositivity against a conjugate formed by a natural octyl disaccharide bound to human serum albumin (NDO-HSA), suggesting the use of IgA as a biomarker for diagnosing and classifying the disease, with potential utility for monitoring the follow-up of household contacts [60].

Some limitations were identified in the study, highlighted by the reduction in the number of individuals who agreed to participate in the project and the clinical evaluations, as well as the progressive loss of the initial population of the COVID-19 serosurvey to the population sample of the leprosy study. The low adherence of individuals to the neurodermatological evaluation makes it impossible to interpret the positivity of antibodies and their association with the diagnosis of leprosy. Stigma, discrimination, and lack of knowledge of the community lead to delayed seeking of care and are factors that contribute to the maintenance of the stigma that leprosy carries, which further increases the late diagnosis, more transmission, and chances of deformities [61]. The Global Leprosy Strategy is based on the pillars of ensuring zero disability and zero stigma related to leprosy through a network of public policy actions, humanized care, and health education aimed at ending discrimination and promoting inclusion [3].

Despite a distribution of cases and individuals throughout the municipality’s territory, the identification of clusters and spatial mapping does not account for the number of people in the sample calculation, which is necessary for an ideal representation of the population of the evaluated region. The absence of spatial differentiation may reflect the relative socioeconomic homogeneity of Ribeirão Preto, the prolonged and predominantly intradomiciliary transmission dynamics of leprosy, which are not fully captured by census tract–level analysis, and the limited number of confirmed cases reducing the statistical power to detect spatial clusters, resulting in a random distribution across the municipality. However, the sample size meets the minimum threshold for conducting Moran’s I analysis, and the inclusion of serological data across multiple census tracts provided complementary information to strengthen our spatial analysis approach despite the small number of clinical cases.

All diagnosed cases and their household contacts were notified for epidemiological surveillance and referred to health units for recommended clinical management. The study presents an initial approach to the application of anti-Mce1A antibodies in a serosurvey, highlighting the need for further studies to track regions considered to be of low endemicity, compare them with areas of greater endemicity, and facilitate the identification of priority groups and hidden endemicity for disease control. However, because not all seropositive individuals underwent clinical evaluation, the interpretation of biomarker responses is limited, and the assays should be viewed as screening and complementary tools that support clinical diagnosis.

## Conclusions

In summary, the diagnosis of leprosy presents a technical and practical challenge, especially in early cases with predominantly neural involvement. Technological development and scientific investment in the area of neglected diseases, such as leprosy, are crucial for controlling the disease as a national public health issue. The search for new laboratory tests with greater sensitivity and accuracy, while maintaining satisfactory specificity, is a proposal to eliminate the main limitations in the laboratory diagnosis of leprosy and detect mild cases. Thus, new biomarkers that achieve the WHO’s goals of identifying initial and infected cases and interrupting bacillary transmission align with the pillars of carrying out research with social impact and contributing to the public health system and mapping. The study reinforces the importance of epidemiological history and the presence of overlapping positive laboratory tests and clinical markers in diagnostic screening for active search strategies of new cases. Furthermore, it plays a role in advancing progress towards Sustainable Development Goal 3 (SDG 3), particularly in the control of neglected tropical diseases such as leprosy.

Thus, the study highlighted the gains from the use of serological screening tools with new biomarkers, reaffirming the importance of health surveillance actions through active search for the detection of oligosymptomatic cases, in early diagnosis with the use of technological platforms for clinical screening. On the other hand, the study demonstrated that IgM anti-Mce1A serology and MaLeSQs^®^ can serve as valuable screening tools for enhancing the efficiency of large-scale active case-finding, maintaining high sensitivity while substantially reducing the number of individuals referred for clinical evaluation, yet still identifying nearly the same number of new cases. Thus, identifying hidden endemic cases in regions considered controlled for leprosy serves as a signal to the municipality, highlighting the need for more comprehensive strategies to search for cases throughout its territory, regardless of sociodemographic markers.

## Supplementary Information

Below is the link to the electronic supplementary material.

Supplementary Material 1

Supplementary Material 2

## Data Availability

All data generated or analyzed during this study are included in this published article [and its supplementary information files].

## Abbreviations

APGL: I IgM anti-phenolic glycolipid-I
COVID-19: Coronavirus Disease-19
Ct: Cycle threshold
DNA: Deoxyribonucleic acid
ELISA: Enzyme-linked immunosorbent assay
FDR: False Discovery Rate
G1D: Physical Disability Grade 1
HHC: Household Contacts
HIV: Human Immunodeficiency Virus
Ig: Immunoglobulin
IPVS: São Paulo State Social Vulnerability Index
IQR: Interquartile Range
LID-1: Recombinant Fusion Protein
LISA: Local Indicators of Spatial Association
LSQ: Leprosy Suspicion Questionnaire
MaLeSQs: Machine Learning for Leprosy Suspicion Questionnaire and Screening
MB: Multibacillary
Mce: Mammalian Cell Entry
mce1A: Mammalian Cell Entry Gene 1A
Mce1A: Mammalian Cell Entry Protein 1A
MDT: Multidrug Therapy
ND-O-BSA: Natural-Octyl Disaccharide Conjugated to Bovine Serum Albumin
NDO-HSA: Natural-Octyl Disaccharide Conjugated to Human Serum Albumin
O.D.: Optical Density
PB: Paucibacillary
qPCR: Real-Time Polymerase Chain Reaction
RLEP: *M. leprae*-Specific Repetitive Element
SARS-CoV-2: Severe Acute Respiratory Syndrome Coronavirus 2
SDG: Sustainable Development Goal
WHO: World Health Organization
