# Supplementary material for: Serum geoepidemiology of leprosy biomarkers in a city-wide COVID-19 survey in Brazil
Source: BMC Infect Dis. 2026 Jan 7;26:258. doi: 10.1186/s12879-025-12483-0 (PMC12870420; doi:10.1186/s12879-025-12483-0)
Supplement: Supplementary file 1 — Supplementary Material 1 [file 12879_2025_12483_MOESM1_ESM.docx]

***SUPPLEMENTARY MATERIAL***

# 1 Supplementary Data

**Global Spatial Analysis (Univariate Moran’s I)**

**Analyzed variable:**

Absolute number of diagnosed cases of leprosy by census tract.

**Results:**

Moran’s I: -0.0059

Expected value (E[I]): -0.0007

Standard deviation: 0.0149

Z-Score: -0.3719

Pseudo p-value: 0.4542

| 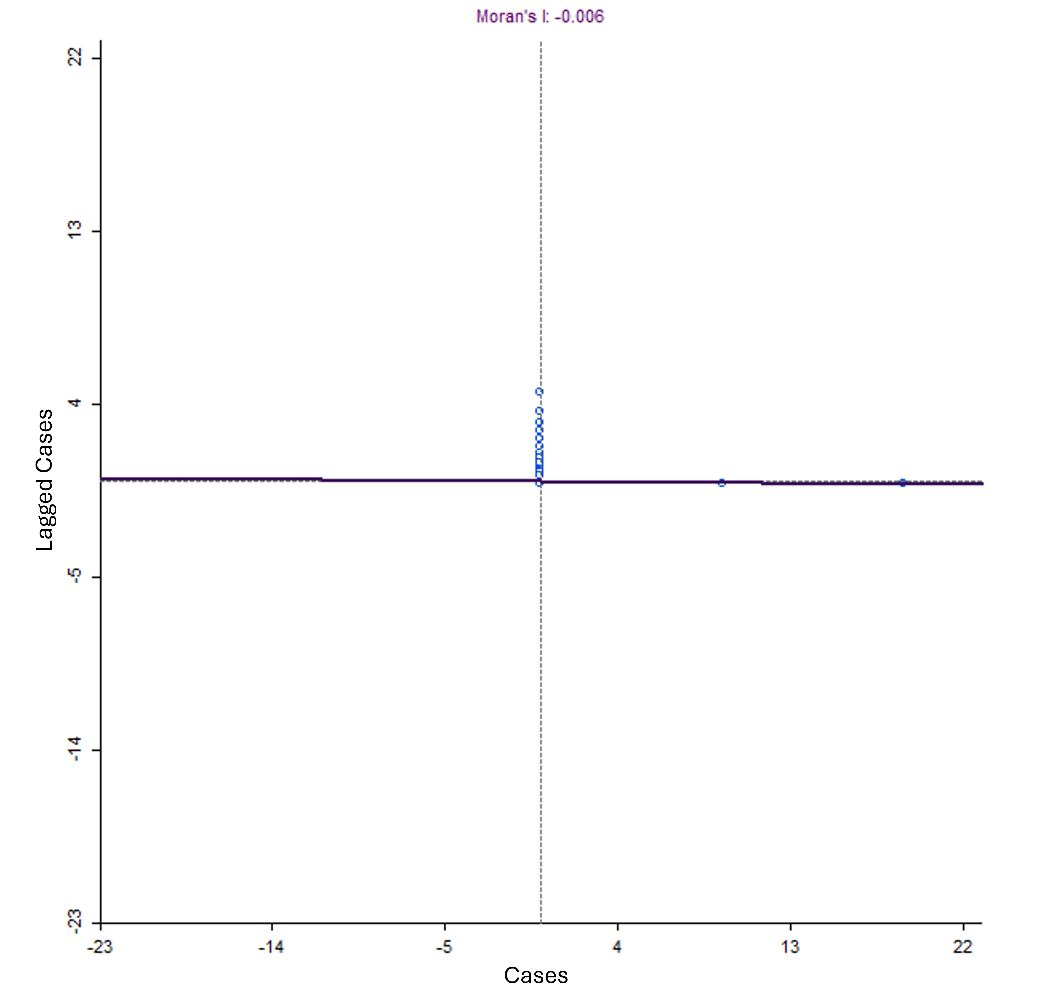 |
| --- |
| **Supplementary Figure 1.** Spatial scatter plot of univariate Moran’s I applied to the number of leprosy cases by census sector. |

| 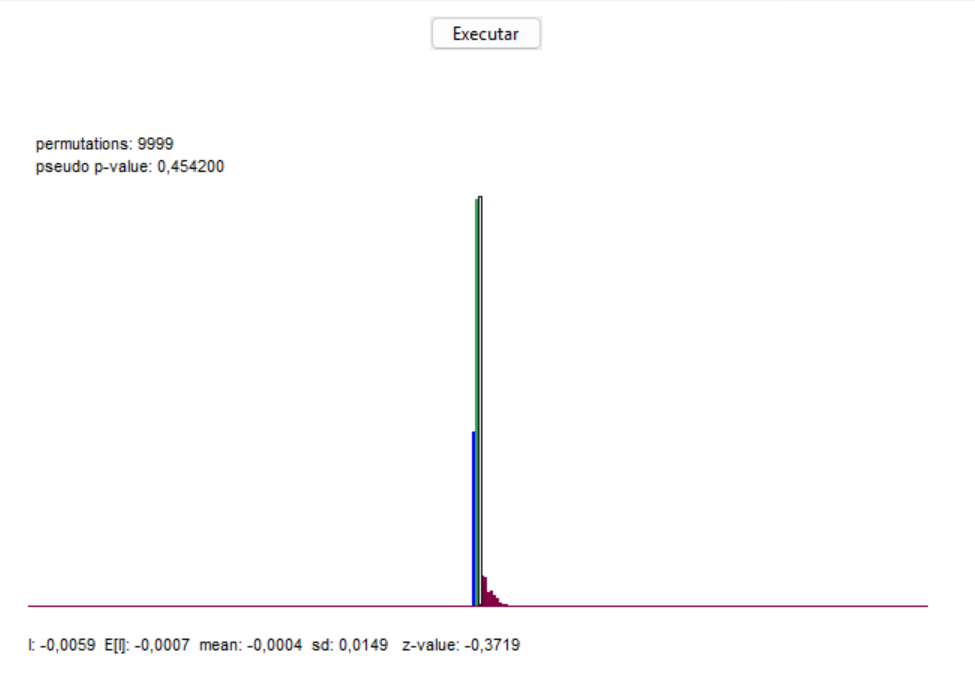 |
| --- |
| **Supplementary Figure 2.** Empirical distribution of Moran’s I permutations (9,999 permutations) for leprosy cases, with observed value, expected value and pseudo p-value. |

**Variable analyzed:**

Number of patients with positive serology for IgM Anti-Mce1A by census sector.

**Results:**

Moran’s I: -0.004

Expected value (E[I]): -0.0007

Standard deviation: 0.0155

Z-Score: -0.0144

Pseudo p-value: 0.4369

| 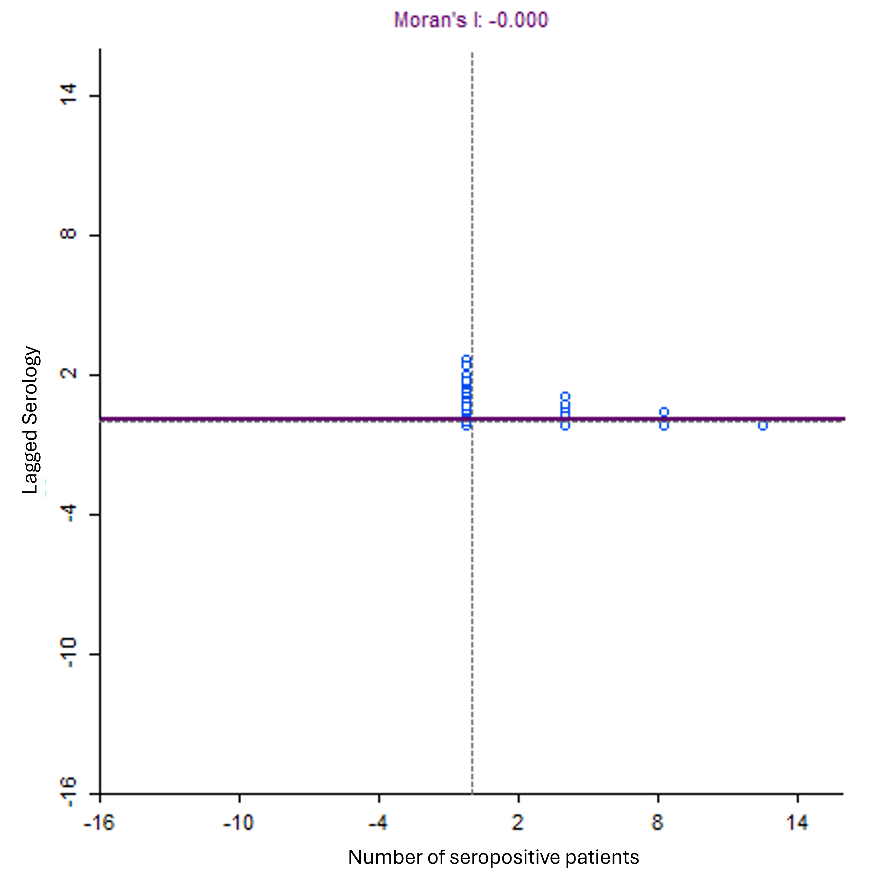 |
| --- |
| **Supplementary Figure 3.** Spatial scatter plot of univariate Moran’s I applied to the number of patients with positive serology for IgM Anti-Mce1A by census sector. |

| 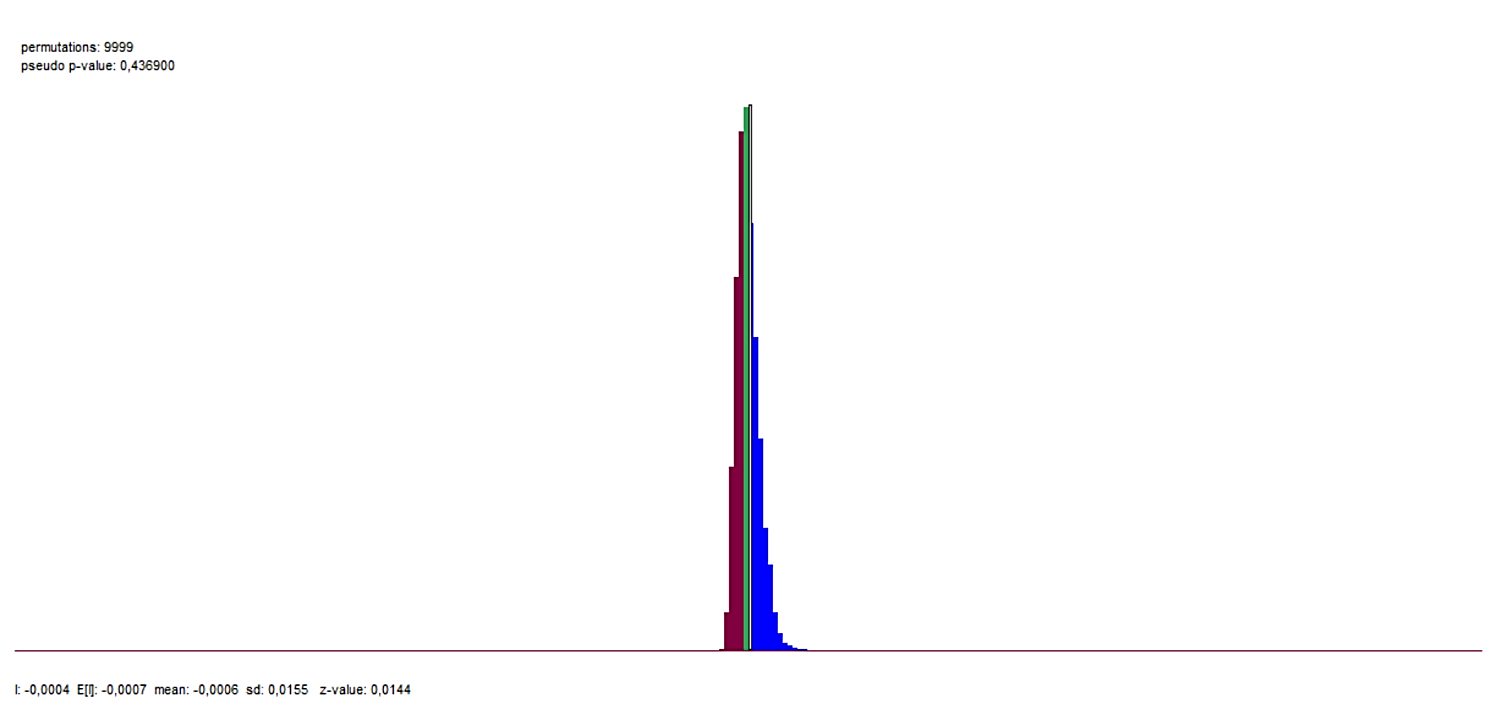 |
| --- |
| **Supplementary Figure 4.** Empirical distribution of Moran’s I permutations (9,999 permutations) for cases with positive serology for IgM Anti-Mce1A, with observed value, expected value and pseudo p-value. |

# 2 Supplementary Data

**Local Spatial Analysis (Univariate LISA)**

**Analyzed variable:**

1. Absolute number of diagnosed cases of leprosy by census sector.

2. Number of patients with positive serology for IgM Anti-Mce1A by census sector.

| 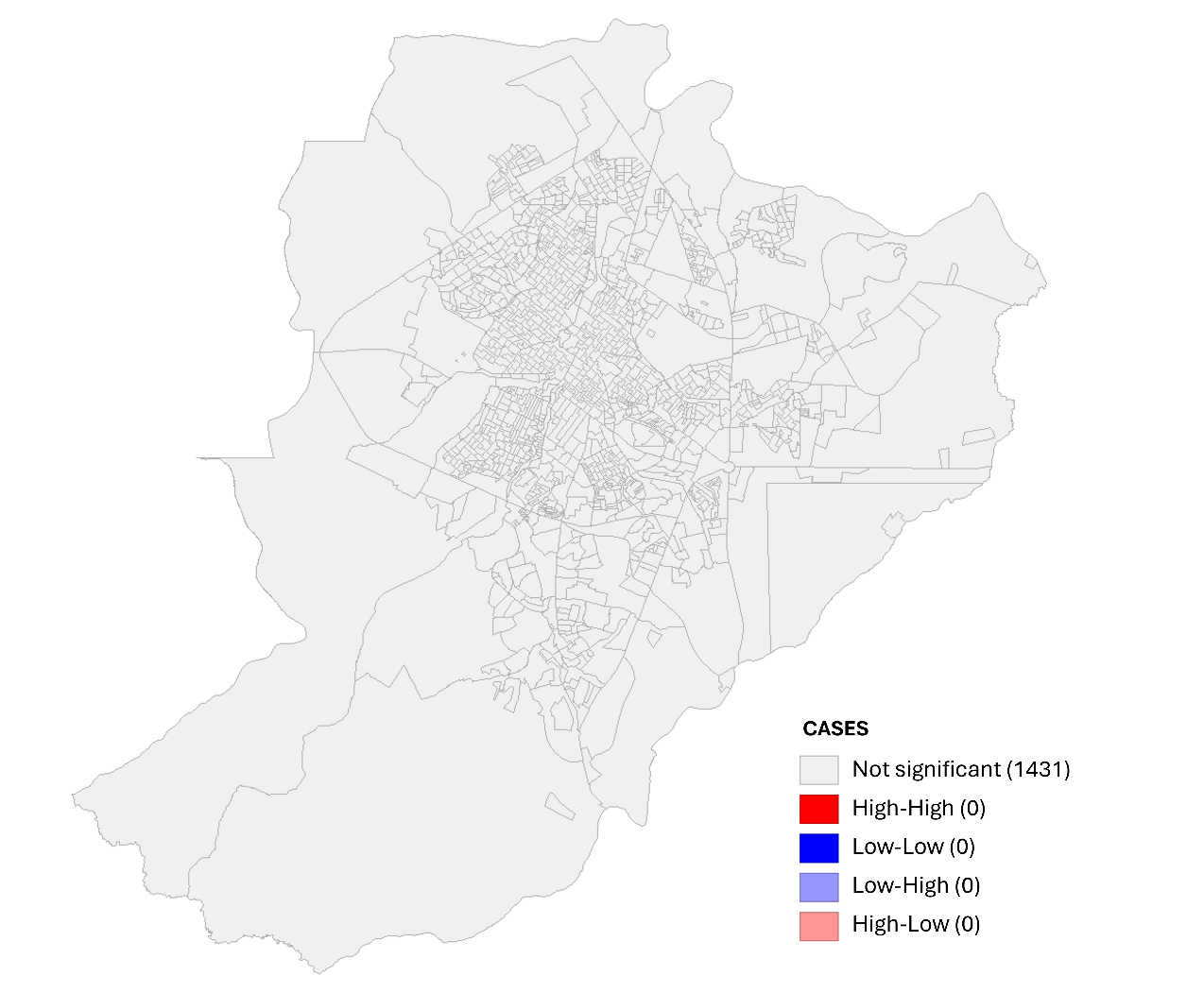 |
| --- |
| **Supplementary Figure 5.** Spatial cluster map (univariate LISA) for leprosy cases after adjustment for False Discovery Rate (FDR), showing absence of local spatial significance. |

| 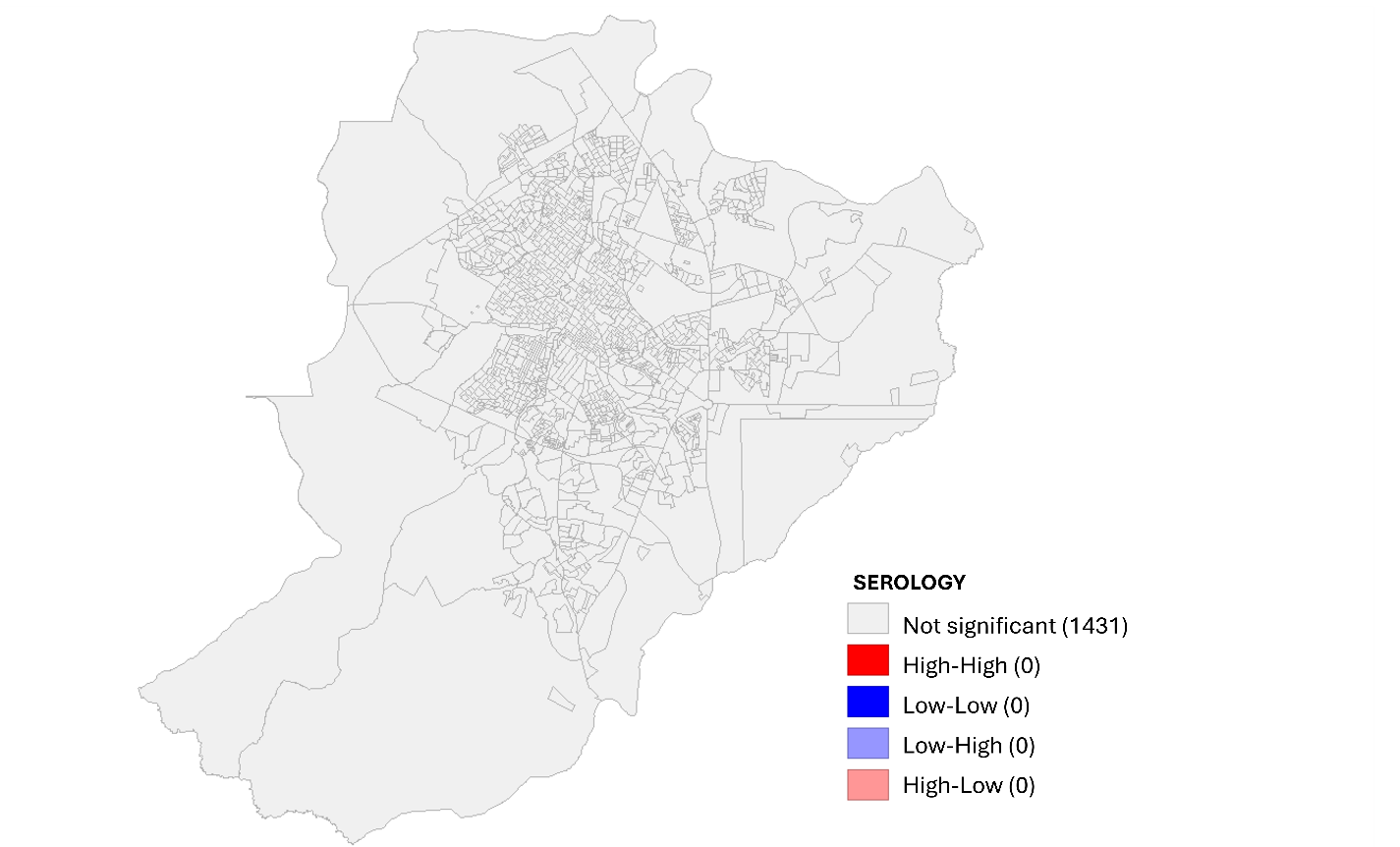 |
| --- |
| **Supplementary Figure 6.** Spatial cluster map (univariate LISA) for patients with positive IgM Anti-Mce1A serology after adjustment for False Discovery Rate (FDR), showing absence of local spatial significance. |

# 3 Supplementary Data

**Bivariate LISA Analysis**

| Supplementary Table 1. Results of the Bivariate LISA Analysis between leprosy cases and IgM Anti-Mce1A serology and sociodemographic variables, with correction for False Discovery Rate (FDR). | | |  |
| --- | --- | --- | --- |
| Sociodemographic variable analyzed | **Variable type** | **Bivariate LISA result with FDR (999 permutations)** | **Moran’s I** |
| Average number of residents in the household | Continuous quantitative | No significant clusters | 0.002 |
| Per capita income in the households | Continuous quantitative | No significant clusters | -0.016 |
| Proportion of households with water supply | Proportional (%) | No significant clusters | 0.003 |
| Proportion of households with sewage system | Proportional (%) | No significant clusters | 0.003 |
| Proportion of households with electricity | Proportional (%) | No significant clusters | 0.002 |
| IPVS classification (São Paulo Social Vulnerability Index) | Categorical ordinal | No significant clusters | 0.003 |
